# Modeling Truncated AR Expression in a Natural Androgen Responsive Environment and Identification of RHOB as a Direct Transcriptional Target

**DOI:** 10.1371/journal.pone.0049887

**Published:** 2012-11-29

**Authors:** Hui-Chi Tsai, David L. Boucher, Anthony Martinez, Clifford G. Tepper, Hsing-Jien Kung

**Affiliations:** 1 Department of Biochemistry & Molecular Medicine, School of Medicine and Cancer Center, University of California Davis, Davis, California, United States of America; 2 Department of Biomedical Engineering, University of California Davis, Davis, California, United States of America; Cedars-Sinai Medical Center, United States of America

## Abstract

Recent studies identifying putative truncated androgen receptor isoforms with ligand-independent activity have shed new light on the acquisition of androgen depletion independent (ADI) growth of prostate cancer. In this study, we present a model system in which a C-terminally truncated variant of androgen receptor (TC-AR) is inducibly expressed in LNCaP, an androgen-dependent cell line, which expresses little truncated receptor. We observed that when TC-AR is overexpressed, the endogenous full length receptor (FL-AR) is transcriptionally downmodulated. This in essence allows us to “replace” FL-AR with TC-AR and compare their individual properties in exactly the same genetic and cellular background, which has not been performed before. We show that the TC-AR translocates to the nucleus, activates transcription of AR target genes in the absence of DHT and is sufficient to confer ADI growth to the normally androgen dependent LNCaP line. We also show that while there is significant overlap in the genes regulated by FL- and TC-AR there are also differences in the respective suites of target genes with each AR form regulating genes that the other does not. Among the genes uniquely activated by TC-AR is RHOB which is shown to be involved in the increased migration and morphological changes observed in LN/TC-AR, suggesting a role of RHOB in the regulation of androgen-independent behavior of prostate cancer cells.

## Introduction

Prostate cancer (CaP) initially presents as an androgen dependent (AD) disease, but frequently progresses to an androgen depletion independent (ADI) or castration-resistant state. As the latter escapes therapies which target the androgen receptor signaling axis, considerable efforts have been made to more thoroughly understand both the transition to and biology of ADI disease. The most representative *in vitro* model of CaP transition from AD to ADI growth is the CWR22R*v1* cell line. Like the AD CaP cell line LNCaP, CWR22R*v1* retains a functional androgen receptor (AR) and, as such, is responsive to the presence or absence of DHT. However, in contrast to LNCaP and more in line with advanced CaP cell lines, CWR22R*v1* is not dependent upon the presence of DHT for growth.

Because of the unique niche it occupies within the collection of CaP cell lines, CWR22R*v1* has been studied extensively within the context of acquisition of ADI growth. As expected, considerable research has focused on the CWR22R*v1* androgen receptor (AR) which has been shown to carry the common H874Y mutation [1] as well as a duplication of exon 3 [2], [3]. We previously reported that CWR22R*v1* and the relapsed CWR22 variant xenograft from which it was derived express an AR with a duplication of exon 3, which is accompanied by a high level of truncated AR. These properties are not present in the original androgen-dependent CWR22 xenograft, and we suggested that the truncated receptor may be responsible for the transition to its androgen-independent state. Using antibodies targeting different regions of AR, we mapped the truncated receptor species to be the N-terminal half of the molecule, consisting of NTD and DBD [2]. Since that initial characterization, the genome of CWR22R*v1* has been found to carry an intragenically duplicated AR locus [4], which may account at least in part for the generation of full-length AR (FL-AR) with a duplicated exon 3 and the wide range of splice variants, although the exact mechanisms remain to be elucidated.

Studies by Libertini et al [5] implicated calpain in the proteolytic cleavage of full length receptor, contributing to some of the truncated receptors. By contrast, the work of Dehm et al [6] suggested AR spliced variants (AR1/2/2b and AR1/2/3/2b) are largely responsible for the generation of the truncated receptors in CWR22R*v1*. Work by Hu et al [7] and Guo et al [8], identified several additional spliced variants and found that AR3/AR-V7, but not AR1/2/2b or AR1/2/3/2b is the predominant species in CWR22R*v1*. Guo et al, went on to develop antibodies against AR3 and conclusively identified the presence of the truncated receptor corresponding to AR3. While the direct demonstration of a cleaved product at the protein level is more challenging than the detection of spliced transcripts, none of the studies have excluded the contributions of truncated receptor generated by proteolytic cleavage. Indeed, Hornberg et al [9] showed that there is a disparity between the amount of truncated receptor and the level of spliced variants. We therefore favor the hypothesis that truncated receptors can be generated through both proteolytic cleavage and alternative splicing.

**Figure 1:**
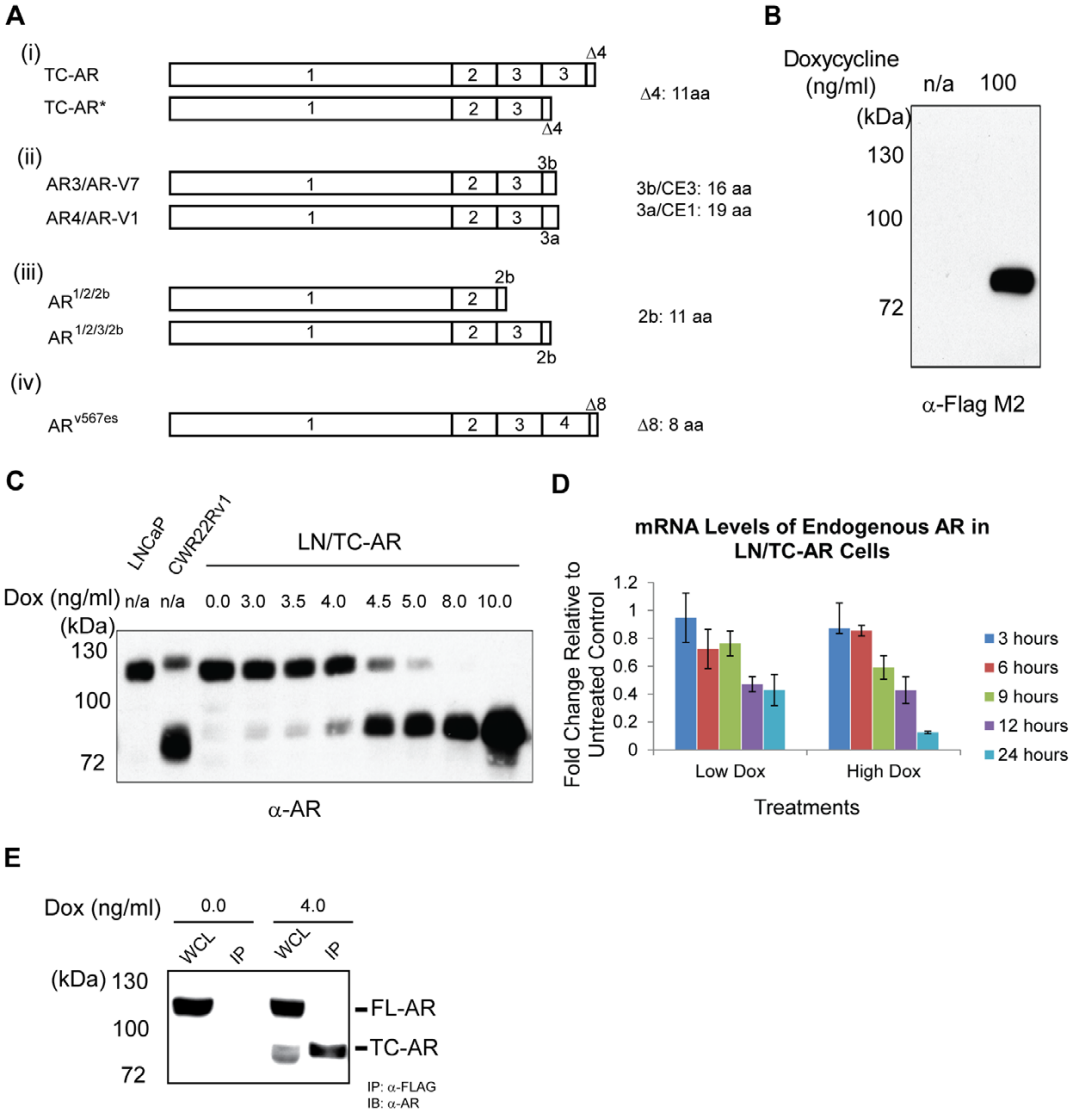
Expression of FLAG-tagged TC-AR in LN/TC-AR cell line suppresses endogenous AR at both transcriptional and translational levels. **A** Schematic structure of prototypic AR truncated variants. (i) Two truncated AR variants used in our study. (ii) AR splice variants from Guo et al 2009 and Hu et al 2009. (iii) AR splice variants from Dehm et al 2008. (iv) AR splice variant from Sun et al 2010. Numbers represent exon numbers. 2b, 3b, CE1, and CE3 represent cryptic exons. The length of translated sequence by cryptic exons is shown at the right. **B** Membrane was probed with α-FLAG M2. Right lane contains 100 µg whole cell lysate harvested from LN/TC-AR cells induced with 100 ng/mL doxycycline for 48 hours. Left lane contains 100 µg whole cell lysate harvested from uninduced LN/TC-AR. **C** LNCaP (LN) and CWR22R*v1* (CWR) lysates serve as control for LN/TC-AR cells which were treated with various concentrations of doxycycline. All lanes contain 100 µg whole cell lysate per lane and the membrane was probed with α-AR(PG-21). **D** LN/TC-AR cells were treated with either Low Dox or High Dox. At designated time points, RNA was isolated and quantitative RT-PCR was performed to determine mRNA levels of endogenous AR and TC-AR. Fold induction is relative to uninduced LN/TC-AR control. **E** Co-immunoprecipitation assay reveals that in LN/TC-AR cells induced with 4 ng/mL doxycycline (Lanes 3&4), TC-AR, but not FL-AR, is precipitated by IP with α-FLAG agarose beads (Lanes 4) indicating that the two AR forms do not heterodimerize. As controls, identically prepared/immunoprecipitated uninduced LN/TC-AR cell lysate is shown in Lanes 1 (WCL) and 2 (IP).

The schematic structure of the prototypic AR truncated variants in our reports as well as in other studies is shown in Figure 1A. Based on the protease cleavage model, the truncated AR would have a duplicated exon 3 and a C-terminal putative calpain cleavage site in the hinge region (Figure 1A (i)). The most abundant and well characterized splice variant is AR3/AR-V7 which has a similar structure except the last 16 C-terminal amino acids are derived from a cryptic exon (CE3) [7], [8], [10]. Another recent study also found a novel human AR splice variant (AR^v567es^) from prostate cancer xenografts with exons 5, 6, and 7 deleted, which is constitutively active in prostate cancer cell lines [11]. There are other splice variants similar to these prototypes, which are present less abundantly in prostate cancer cell line or tissues, and of which the protein products have yet to be identified [10], [12], [13]. These variants all lack the major part of the ligand binding domain and their activities are ligand-independent. Although in FL-AR the hinge domain is required for nuclear localization [14], most reported splice variants are lacking the hinge region (and the nuclear localization signal encoded within it). However, as in the case of AR3/AR-V7, this deletion may be compensated for by a cryptic exon rich in lysine residues [12].

Most of the previous studies on truncated receptors have focused on their ability to transactivate androgen-responsive genes and to induce androgen-independent growth [6]–[8]. These studies were carried out primarily either in androgen-insensitive, AR negative cells, or in androgen-responsive cells with a high level of FL-AR, which preclude the analysis of isolated TC-AR in its natural androgen-responsive background. The presence of FL-AR apparently can affect the function of the spliced variants in a cell context dependent manner [10], [15]. Furthermore, most of the biological studies utilize clones derived from transduction or transfection of cDNA carrying truncated receptors, which may have acquired properties different from the parental cells. As a potentially more attractive model, we present here the establishment of LN/TC-AR, a novel cell line derived from the androgen dependent LNCaP parental line in which expression of a C-terminally truncated AR (TC-AR) is regulated and titratable by doxycycline and automatically down modulates the expression of the endogenous FL-AR. As such, when uninduced, the main AR species is the FL-AR of LNCaP, whereas upon induction, TC-AR of CWR22R*v1* predominates. The biological and transactivational properties of FL-AR and TC-AR can thus be studied in exactly the same genetic and cell background.

To demonstrate the utilization of this cell line, we report the presence of autoregulation of AR expression levels, acquisition of ADI growth, and changes in cell shape and migration following induction of TC-AR. We also extend upon reporter assays involving C-terminally truncated AR forms to show occupancy at an AR regulated promoter and transcriptional activation of an AR regulated gene. Using microarray and qRT-PCR, we report on the common and unique genes regulated by TC-AR and DHT-bound endogenous AR. Lastly, while its effect is not directly involved in ADI growth, we identify *RHOB*, a gene we show to be upregulated by TC-AR, but not by DHT-bound endogenous FL-AR, to be a possible effector of morphological and migration differences during the transition to ADI disease. As the AR forms in these cell lines represent the minimal common domains shared by all functional ligand-independent AR, the data generated by this study will likely be applicable to similarly truncated receptors.

## Materials and Methods

### Plasmid Construction

TC-AR was PCR amplified (forward primer: 5′-ggatccaatgtaagtgcagttagggctggg-3′; reverse primer: 5′-ctcgagtcatagtttcagattaccaagtttcttcagcttcc-3′) from cDNA derived from CWR22R*v1*, TOPO cloned and verified for sequence accuracy. The insert was then excised from the cloning vector using *BamHI* and *XhoI* restriction sites and ligated into a similarly digested modified form of pLenti4/TO/V5-DEST (Invitrogen). Subcloning was done such that TC-AR was placed immediately downstream and in frame with sequence encoding the FLAG epitope to produce the lentiviral expression plasmid pLenti4/TO/FLAG-TC-AR.

### Cell Lines

LNCaP and 293T cells were obtained from ATCC and cultured in RPMI or DMEM, respectively, both of which were supplemented with 10% FBS and 1% PSG. All cells were cultured at 37C in the presence of 5% CO_2_ in air. Stable cell lines derived from the parental LNCaP line were each established following lentiviral transduction and drug selection of stable transductants using the ViraPower tRex system (Invitrogen) according to manufacturer's instructions. Briefly, 293T cells were co-transfected with each of three helper plasmids along with the appropriate expression or knockdown plasmid. Lentiviral particle-containing supernatant was then harvested 48-hours post transfection, filtered and applied to the appropriate parental cells which were then cultured in the presence of blasticidin (10 ng/mL; Invitrogen), zeocin (100 ng/mL; Invitrogen) or puromycin (0.5 ug/mL; Invitrogen). Drug resistant clonal populations were then isolated, expanded and analyzed for gene expression or gene knockdown by western blot. A summary of cell lines reported here and their derivation (parental line; expression plasmid; drug selection) is as follows: LNCaP/TR (LNCaP; pLenti6/TR; blasticidin), LN/TC-AR (LNCaP/TR; pLenti4/TO/FLAG-TC-AR; zeocin) and LN/TC-AR/shR-RHOB (LN/TC-AR; pLKO.1/shR-RHOB; puromycin). Lentiviral plasmids encoding shRNA sequence targeting *RHOB* were purchased from Open Biosystems. Plasmid name with corresponding mature anti-sense region is pLKO.1/shRHOB; AACTCGTCCTTACTGAACACG.

### Western Blot Analysis

Cells were lysed in RIPA buffer supplemented with Complete Protease Inhibitor Cocktail (Roche). Nuclei and cellular debris were removed by centrifugation at 14,000×g for 15 min. at 4 C. Protein concentration was then assayed using the BCA Protein Assay Reagent (Pierce). 100 µg samples were then separated by denaturing SDS-PAGE and transferred to PVDF membranes (Immobilon). Membranes were blocked for 90 min. in 5% NFDM in TBST. Primary antibodies α-FLAG M2 (Sigma-Aldrich), α-AR (PG-21) (Millipore), α-actin (Sigma-Aldrich) and α-RHOB (Santa Cruz) were diluted in 5% NFDM in TBST according to manufacturer's recommendations and incubated with the membranes overnight at 4 C. Membranes were then washed twice with TBST for 15 min. and incubated with either α-mouse or α-rabbit secondary antibody conjugated to HRP (Santa Cruz) and diluted 1∶5000 in 5% NFDM in TBST for 90 min. at RT. Membranes were washed twice with TBST and developed with Supersignal (Pierce) and exposed to Bio-Max X-Ray film (Kodak).

### Co-immunoprecipitation Assay

LN/TC-AR cells were grown in RPMI 1640 supplemented with 10% FBS/1% PSG and induced with 4 ng/mL doxyxycline for a period of 48 hours with uninduced cells serving as a negative control. Cells were lysed in Pierce's IP lysis buffer supplemented with Halts protease and phosphatase inhibitors (Pierce Biotechnology) and samples were centrifuged (15 krpm; 10 m; 4 C) to clear lysate. Supernatant was then transferred to a fresh 1.5 mL centrifuge tube and concentration was determined by BCA assay. An aliquot of cell lysate was reserved as an input control and 500 µg lysate was mixed with 50 uL of a 50% slurry of α-FLAG M2 agarose beads (Sigma Aldrich) and incubated overnight at 4 C with constant rotation. Beads were washed 3× with 1 mL Pierce's IP buffer and eluted with 30 µL 6× sample buffer. Samples were resolved on a 6% polyacrylamide gel and transferred to a PVDF membrane. Membranes were probed with α-AR(PG-21) followed by HRP-conjugated α-Rabbit developed with SuperSignal and imaged on a Cell Biosciences western blot imager.

### Luciferase Assay

LN/TC-AR cells were seeded to 24-well plates at a density of 60 K/well and incubated overnight at 37 C. The following day, cells were co-transfected with pPSA6.0-luc (180 ng/well) and pH 48-ren (20 ng/well) using Effectene Transfection Reagent (Qiagen) and treated with either DHT or various concentrations of doxycycline. Forty-eight hours post-transfection/treatment, cells were lysed and assayed for luciferase and renella production using the Dual-luciferase Assay Kit (Promega) as recommended by the manufacturer on an EG&G Berthold LB96V MicroLumatPlus microplate luminometer (Perkin-Elmer-Wallace, Inc.). All sample groups were completed in triplicate (mean values are reported) and have been normalized for transfection efficiency using the renella relative light units (RLU) acquired for each sample.

### Immunostaining

LN/TC-AR cells were plated to 6-well plates with one slide in each well and were hormone-depleted for 3 days then treated with 4.5 ng/mL doxycycline treatment or vehicle only as control for 24 hours. Slides were washed with 1× PBS twice and fixed in 4% paraformaldehyde at 4°C for 30 minutes. After three washes with 1× PBS, slides were permeabilized with 1% Triton X-100 in 1× PBS followed by 1% NP-40 in 1× PBS for 10 minutes. Slides were blocked with 2% BSA in TBST for 30 minutes then washed 3 times with 1× PBS. Anti-FLAG M2 antibody was diluted 1∶50 in 2% BSA in TBST and incubated with slides for one hour at room temperature (RT). Slides were then washed with 1× PBS 3 times and incubated with Alexa Fluor 488 goat-α-mouse secondary antibody (Invitrogen) at RT for one hour. Slides were washed 3 times with 1× PBS and mounted with SlowFade and DAPI (Invitrogen). Images were acquired with an Olympus fluorescent microscope using appropriate filter sets.

### Cell Growth Assays (Cell Counts & MTT)

#### Cell count assay

Cells were plated to 6-well plates at a density of 2×10^5^ cells/well in RPMI1640 supplemented with 10%CDT-FBS, 1%PSG. After 24 hours, cells were treated with DHT 1 nM, Dox 4.5 ng/mL, Dox 20 ng/mL and vehicle only as control. Medium was refreshed every 72 hours. Individual treatments were in duplicate. At the reported time points, cells were washed gently with PBS and trypsinized 2–3 minutes at RT. Total cells per well were determined via Countess® Automated Cell Counter (Invitrogen) according to the manufacturer's protocol.

#### MTT assay

Cells were plated to 24-well plates at a density of 20 K cells/well in RPMI1640 supplemented with 10%CDT-FBS, 1%PSG. After 24 hours, cells were treated with DHT 1 nM, Dox 4.5 ng/mL, Dox 20 ng/mL and vehicle only as control. At the designated time points, 30 µL MTT labeling reagent was added to each well and incubated 4 hours at RT. Following the four hour incubation, 300 µL of the Solubilization Solution was added to each well and the plates were incubated overnight. The following day, absorbance of the formazan product was measured at A570 on a microplate reader.

### Cell Migration Assays

Cell migration assay kit (ECM 509) was purchased from Millipore (CHEMICON) and the commercial protocol from Millipore was followed. Briefly, cells were incubated in RPMI1640 media supplemented with 1%PSG only for 24 hours. 3×10^5^ cells were plated into each insert in RPMI1640 with 0.5% BSA and treated with DHT 1 nM, Dox 4.5 ng/mL, Dox 20 ng/mL and vehicle only as control. Individual treatments were completed in duplicate. 500 µls of RPMI1640 supplemented with 10% FBS was added to bottom chambers and cells were incubated for 48 hours. Cells/media from the top side of each insert were removed and inserts were incubated in 225 µls of Cell Detachment Solution for 30 minutes at 37°C. 75 µL of Lysis Buffer/CyQUANT® GR Dye Solution was added to each well containing Cell Detachment Solution and inserts were incubated in it for 15 minutes at room temperature. 200 µL of each mixture was added to one well of a 96-well plate and fluorescence absorbance was measured by a fluorescence plate reader with a 480/520 nm filter set.

### Microarray

LN/TC-AR cells were treated with DHT 1 nM, Dox 4.5 ng/mL, Dox 20 ng/mL and vehicle only as control in 10%CDT media for 24 hours after 3 days in 10%CDT media. Total RNA was extracted by using QIAshredder and RNeasy Mini Kit and following the protocols from QIAGEN. Two independent experiments were performed and RNA samples were submitted to the UC Davis Cancer Center Gene Expression Resource facility. Microarray analysis was done using Affymetrix Human Genome U133 Plus 2.0 arrays (Affymetrix, South San Francisco, CA). Analysis of the expression data from the Dox 4.5 ng/mL-treated cells, Dox 20 ng/mL-treated cells and DHT-treated cells versus the vehicle-treated cells was conducted using DNA-Chip Analyzer software. Filter criteria are p≤0.05, signal log ratio ≥0.6 (1.5fold) and present (P) for detection. Gene Ontology (GO) analysis was performed by DAVID Functional Annotation tools (http://david.abcc.ncifcrf.gov/home.jsp) and Gene Set Analysis Toolkit [16].

### qRT-PCR

RNA was isolated as described in Microarray section. Complementary DNA synthesis was done using SuperScript® III Reverse Transcriptase Kit (Invitrogen) according to the manufacturer's protocol. 2 ng cDNA was amplified by iQ5 iCycler thermal cycler (Bio-Rad) and monitored by SYBRGreen (Invitrogen) for real time PCR. Threshold cycle values were normalized against actin or GAPDH. Individual samples were performed in triplicate and converted to relative gene expression using QGene96 software (http://www.gene-quantification.de/download.html#qgene). Sequences for each primer set are in Table S3.

### Chomatin immunoprecipitation (ChIP)

ChIP assays (2×10^7^cells/assay) were performed following the University of California Davis Genome Center ChIP protocol (http://genomics.ucdavis.edu/farnham). The primary antibodies used in the assays were α-FLAG M2 antibody (Sigma) and α-RNA polymerase II 8WG16 monoclonal antibody (Covance). *KLK3* promoter primer sequences are: 5′-TCTGCCTTTGTCCCCTAGAT-3′ (forward) and 5′-AACCTTCATTCCCCAGGACT-3′ (reverse) [17].

### ChIP to chip analysis

ChIP assays (2×10^7^cells/assay) were performed following the University of California Davis Genome Center ChIP protocol (http://genomics.ucdavis.edu/farnham). The primary antibody used in the assays was α-FLAG M2 antibody (Sigma-Aldrich). LN/TC-AR cells were treated with 10 ng/mL doxycycline for 24 hours after three days in RPMI supplemented with 10% charcoal dextran treated (CDT) fetal bovine serum and 1% PSG. Two independent ChIP experiments were performed and α-FLAG M2 antibody was used for detecting occupancy of FLAG-tagged TC-AR on chromatins. One total input, one IgG control and two LN/TC-AR ChIP samples were collected and sent to the UCD Cancer Center Gene Expression Resource Facility for hybridization [18]. Data analysis was performed with CisGenome software [19]. TC-AR binding regions were identified by comparison to total input control as well as IgG control using the TileMap peak detection tool [20]. Genomic locations of binding peaks were visualized in the CisGenome browser.

## Results

### Titration of doxycycline induction yields a physiologically relevant level of TC-AR expression in the newly established LN/TC-AR cell line

LN/TC-AR is a newly developed cell line derived from the parental LNCaP line in which a truncated form of the androgen receptor (TC-AR) is expressed following doxycycline induction (Figure 1B). Titration of doxycycline levels showed that TC-AR expression was maximal when cells were cultured in complete media supplemented with 10 ng/mL doxycycline (data not shown). A second, more focused titration showed that a physiologically relevant level of TC-AR expression (as defined here by similarity to AR expression in the CWR22R*v1* cell line) was achieved when cells were cultured in complete media supplemented with 4.5 ng/mL doxycycline (Figure 1C). In subsequent studies involving this cell line, induction of TC-AR with 4.5 ng/mL doxycycline (Low Dox) is used to approximate physiological levels of expression while increased doxycycline concentrations (High Dox) are used to induce “overexpression” of TC-AR.

### Induction of exogenous AR causes a concomitant decrease in endogenous AR protein and mRNA levels

Immediately apparent in the doxycycline titrations is the inverse relationship between the induced exogenous TC-AR and endogenous FL-AR protein levels (Figure 1C). In order to investigate whether this effect is limited to post-translational regulation or is also observed at the transcriptional level, real time quantitative reverse transcriptase PCR (qRT-PCR) analysis of endogenous FL-AR in LN/TC-AR was performed. Following induction of TC-AR with Low Dox, a progressive decrease was observed in endogenous FL-AR mRNA at 3, 6, 9 & 12-hour time points after which FL-AR mRNA levels appeared to stabilize at approximately 40% that of normal uninduced cells (Figure 1D). A similar pattern was observed in cells induced with High Dox with endogenous FL-AR mRNA levels decreased to 12% of control at the 24-hour time point. Under the latter conditions FL-AR protein was undetectable by western blot analysis. While we can never exclude the expression of FL-AR in these cells, its contribution should be minimal.

### FL-AR and TC-AR do not form a heterodimer in LN/TC-AR

As heterodimerization of a similarly truncated AR form (AR^v567es^) and FL-AR has been reported [11], we attempted a co-immunoprecipitation of TC-AR and FL-AR using induced LN/TC-AR lysates (Figure 1E). To avoid the significant decrease in FL-AR expression following induction with greater than 4 ng/mL doxycycline (Figure 1C), this assay was completed with lysates harvested from LN/TC-AR induced with 4 ng/mL doxycyline. As expected, IP with α-FLAG M2 agarose beads precipitated the FLAG-tagged TC-AR; however, co-precipitation of FL-AR was not observed indicating that TC-AR does not form a heterodimer with FL-AR in the LN/TC-AR cell line.

### TC-AR is transciptionally active in the absence of DHT

**Figure 2:**
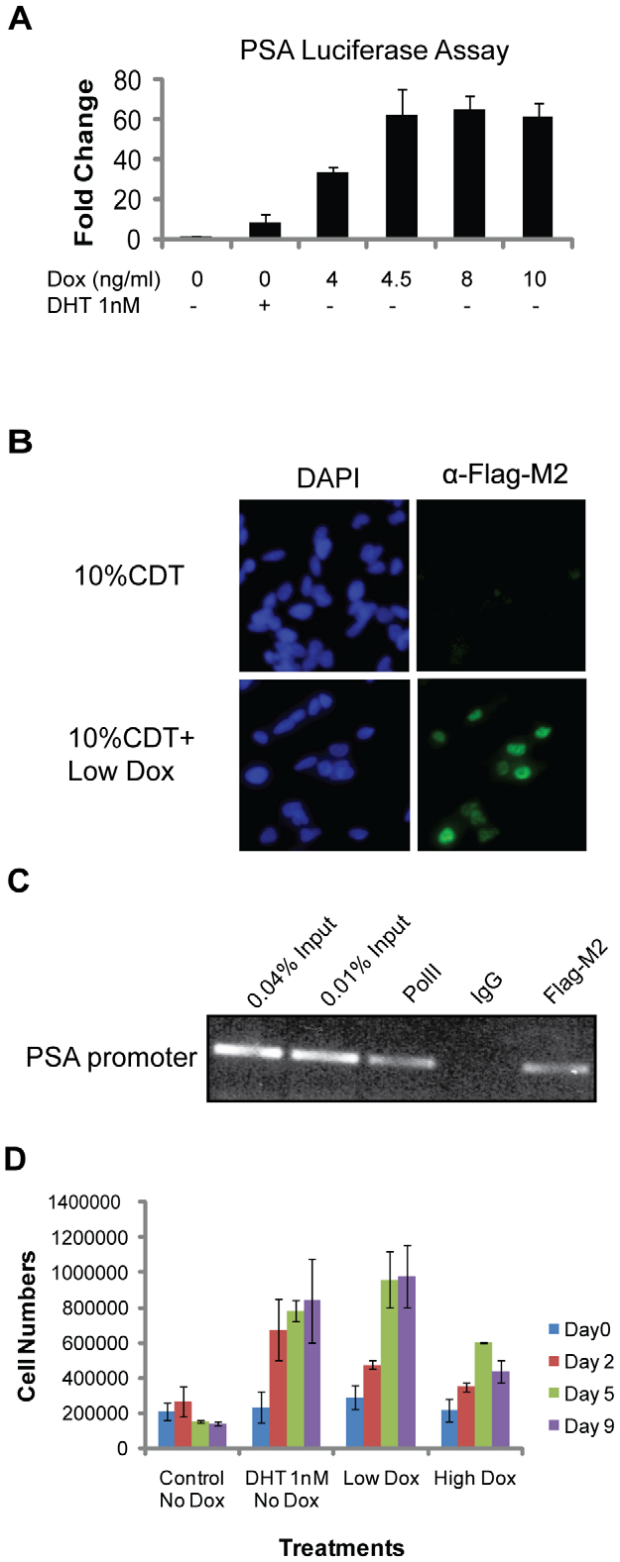
TC-AR is transcriptionally active in the absence of DHT and confers ADI growth *in vitro*. **A** Luciferase assay showing DHT-independent transcription of a transiently transfected AR regulated promoter following induction of TC-AR in LN/TC-AR. LN/ TC-AR cells were co-transfected with pPSA6.0-luc and pH 48-ren in hormone depleted media and treated with either low concentrations of doxycycline, DHT 1 nM or vehicle as control for 24 hours. Fold induction is relative to untreated control. **B** Immunostaining of LN/TC-AR shows androgen independent nuclear localization of TC-AR (right). Cells were counterstained with DAPI to identify nuclei (left) and images were acquired with an Olympus fluorescent microscope using 20× magnification. **C** Chromatin Immunoprecipitation (ChIP) showed the recruitment of AR and RNA polymerase II to the *KLK3* promoter. LN/TC-AR cells were pre-cultured in androgen depleted medium for 72 hours then treated with Low Dox for 24 hours. Anti-FLAG M2 (for FLAG-tagged TC-AR) and α-RNAP2 antibody were used in separate aliquots to immunoprecipitate cross-linked protein and DNA. **D** Cell count assay showing the growth of LN/TC-AR in hormone depleted media following treatment with 1 nM DHT, Low Dox, High Dox or vehicle as control.

In order to examine the ability of TC-AR to facilitate transcription at an AR-regulated promoter, a luciferase assay using the full-length PSA promoter was completed. Immediately following co-transfection of pPSA6.0-luc and pH 48-ren reporter plasmids, expression of TC-AR in LN/TC-AR was induced with various concentrations of doxycycline. Transfected, but uninduced, LN/TC-AR cells treated with either 1.0 nM DHT or vehicle (EtOH) serve as positive and negative controls, respectively. Luciferase production (dependent upon activity of the upstream PSA promoter) was found to be significantly increased in all doxycycline-treated samples relative to untreated control (Figure 2A). Furthermore, transcriptional activity measured for each of the TC-AR expressing samples was three to seven fold higher than that found in the uninduced DHT-treated control in which luciferase production is controlled solely by DHT-bound endogenous AR.

### TC-AR localizes to the nucleus and is able to bind androgen response elements (AREs) in chromatin in the absence of DHT

In order to observe localization of TC-AR, immunostaining of LN/TC-AR was completed. Contrary to endogenous AR which has been shown to remain in the cytoplasm in the absence of DHT, TC-AR localized predominantly to the nucleus following induction with Low Dox (Figure 2B). Chromatin immunoprecipitation (ChIP) assay was performed to assess binding of TC-AR to the AR-regulated *KLK3* promoter (Figure 2C). Occupancy of the *KLK3* promoter by TC-AR following doxycycline induction of LN/TC-AR cells was observed. Unlike wild-type AR, DHT was not required for the binding of TC-AR to the *KLK3* promoter [17]. RNA polymerase II was also found at the *KLK3* promoter thus demonstrating the transcriptional activation of an endogenous androgen regulated gene by TC-AR in the absence of DHT.

### Induction of TC-AR in the LN/TC-AR is sufficient for ADI growth

In order to test whether expression of TC-AR could result in androgen independent growth of the normally androgen dependent LNCaP cell line, a cell count assay was performed (Figure 2D). Uninduced LN/TC-AR cells cultured in 10% charcoal dextran treated FBS (CDT-FBS) in the presence or absence of 1.0 nM DHT serve as “normal” and “androgen depleted” controls, respectively. As expected, culture in androgen depleted medium caused an inhibition of cell growth while those cells grown in normal conditions proliferated as expected. Following induction of TC-AR with Low Dox, LN/TC-AR cells grew in the absence of DHT. Induction with High Dox exhibited a similar phenotype of androgen independent growth, albeit to a lesser degree.

### Cell shape and motility of LN/TC-AR is influenced by the level of TC-AR expression

**Figure 3:**
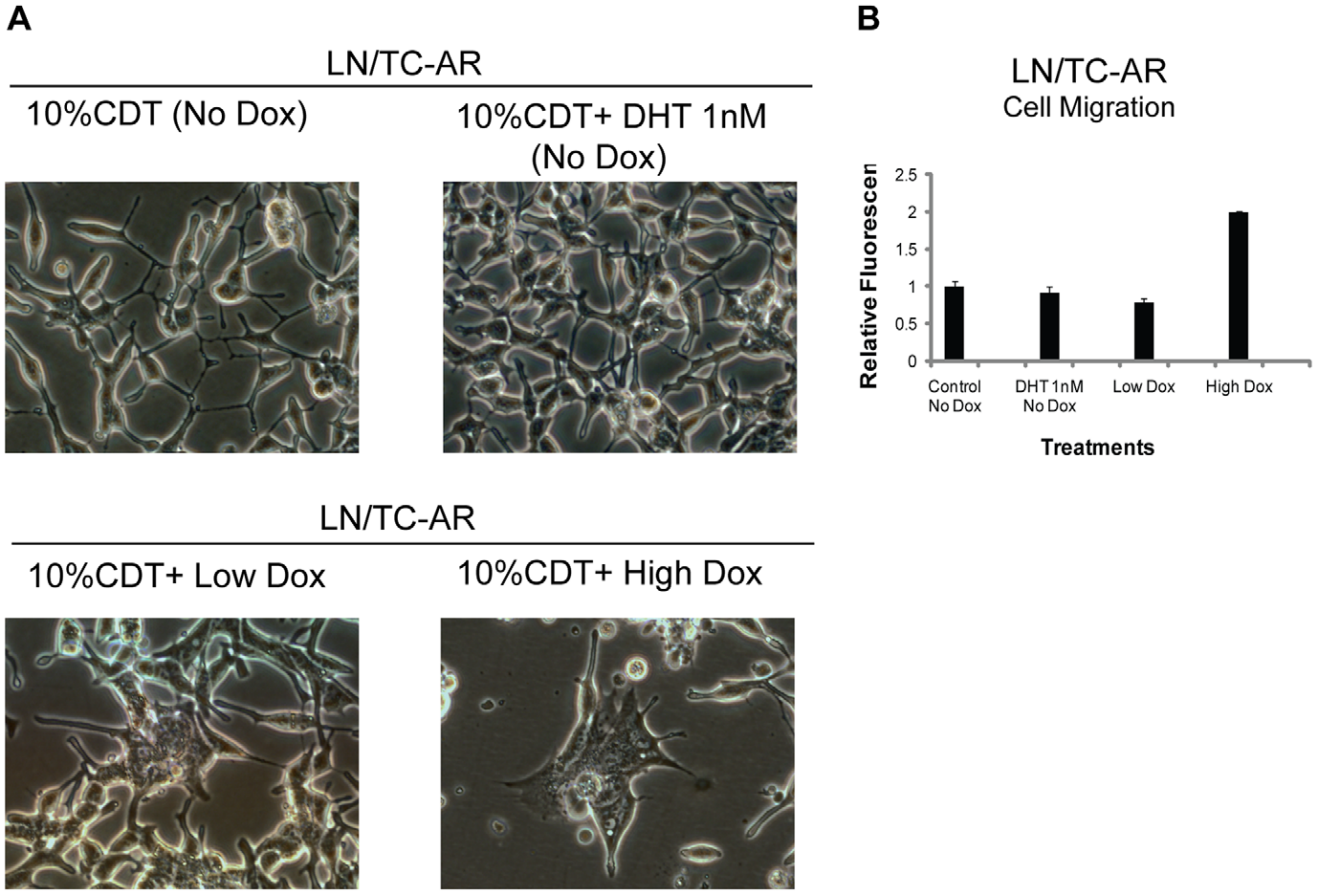
Cell shape and motility change of LN/TC-AR under different dox treatments. **A** LN/TC-AR cells were grown in the presence of hormone depleted media and treated with various concentrations of doxycycline or 1 nM DHT. CWR22R*v1* cells were grown in RPMI supplemented with 10% FBS. At 48-hours post-treatment representative images of each sample group were acquired. **B** LN/TC-AR cells were pre-cultured in serum free media (SFM) for 24 hours then seeded to migration chambers with various treatments in the presence of SFM for an additional 48 hours after which time fluorescence was detected. Fold induction is relative to untreated control.

In addition to the biochemical properties of TC-AR described above and growth characteristics following its induction in LN/TC-AR, cell shape changes were also observed. LN/TC-AR cells were cultured in media containing 10% CDT-FBS and supplemented with 1 nM DHT, Low Dox, High Dox or vehicle (EtOH) only. Representative images were taken of each culture 48-hours post-treatment (Figure 3A). As has been previously described for the parental LNCaP cell line [21], culture in androgen depleted medium (10% CDT-FBS) stimulates the extension of what has been described as “neuritic processes” in uninduced LN/TC-AR. Culture of LN/TC-AR in the presence of Low Dox decreases this “branching” morphology and, in fact, the cell shape is quite similar to uninduced LN/TC-AR grown in the presence of 1 nM DHT. However, induction of TC-AR with High Dox causes a significant change in cell shape in that the characteristic slender cell body normally associated with LNCaP is no longer present. This effect was also observed in cells induced with Low Dox; however, not until approximately six days post-induction (Figure S1). Also observed in LN/TC-AR cells induced with High Dox was a two-fold increase in cell motility relative to uninduced LN/TC-AR (Figure 3B). This increased motility was not observed in LN/TC-AR grown in the presence of 1 nM DHT or Low Dox for the same period of time.

### Identification and validation of TC-AR target genes

**Figure 4:**
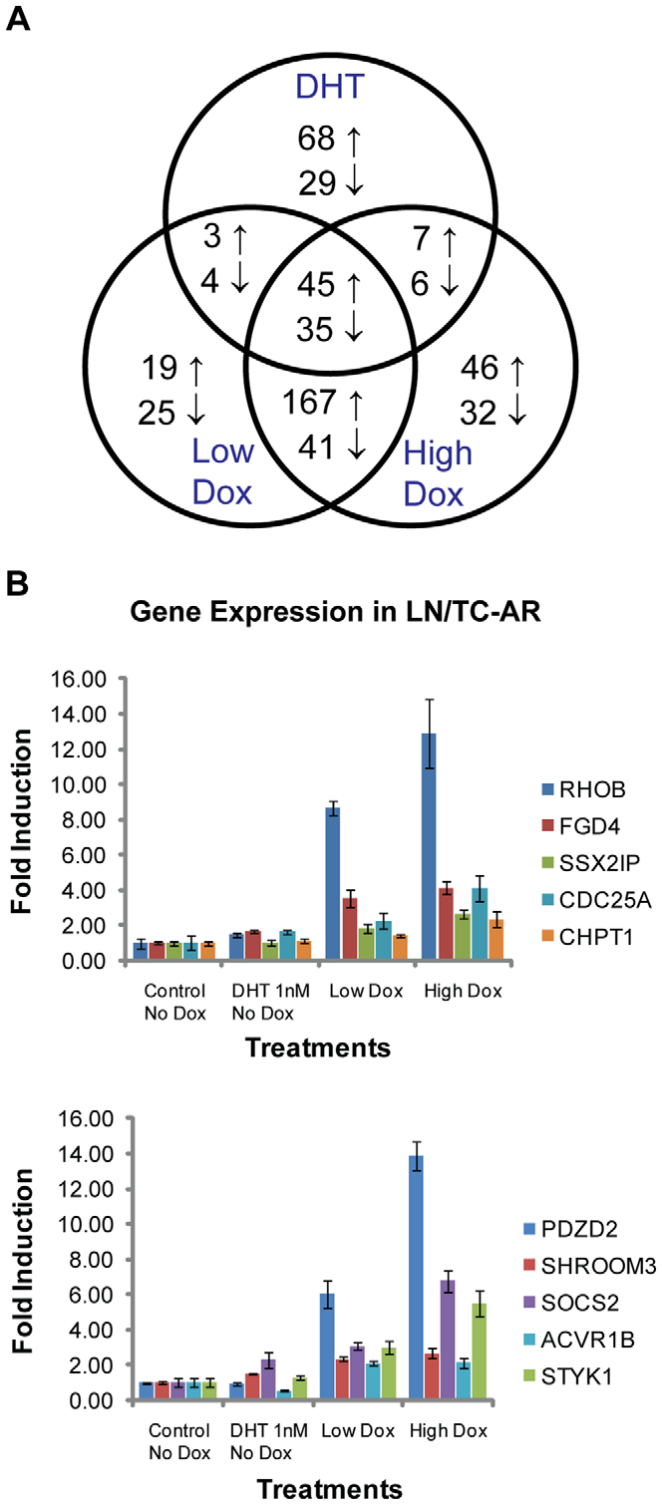
Comparison of gene expression in LN/TC-AR following induction of TC-AR or treatment with DHT. **A** Venn diagram of microarray analyses of LN/TC-AR cells treated with 1 nM DHT or different concentrations of doxycycline for 24 hours post serum starvation. Numbers indicate genes that were commonly or uniquely upregulated (↑) or downregulated (↓) following the various treatments. **B** LN/TC-AR cells were treated with Low Dox, High Dox, 1 nM DHT, or vehicle in hormone depleted media for 24 hours. Quantitative RT-PCR was performed for the selected genes. Fold induction is relative to values obtained in untreated control using identical gene specific primers.

Genome-wide microarray expression profiling with human genome Affymetrix Human Genome U133 Plus 2.0 arrays was completed to compare gene regulation by induced TC-AR and DHT-bound endogenous AR. Analysis showed that there were 197 genes with differential expression following treatment with 1.0 nM DHT, 339 genes following treatment with Low Dox and 379 genes following treatment with High Dox (filter criteria: p≤0.05, signal log ratio ≥0.6 and present (P) for detection). There were 45 genes commonly up-regulated and 35 genes commonly downregulated by each of the three treatments (Figure 4A). Some well-known AR-responsive genes, such as *KLK2*, *KLK3*, *KLK4*, *FKBP5* and *TMPRSS2*, were among the commonly up-regulated genes, which showed that TC-AR shared some common biological influence with endogenous AR (Table S1). However, greater than half of upregulated genes identified in the Low Dox group overlapped with upregulated genes in the High Dox group but not the DHT treatment group.

Based on the microarray data, we acquired a list of genes which were upregulated by both Low Dox and High Dox treatment groups (Table S2). To confirm the microarray results, qRT-PCR was performed on several of these genes. Each of the genes tested showed higher expression in doxycycline treatment groups relative to untreated controls (Figure 4B). Microarray analysis of the expression levels of the TC-AR selectively upregulated *PDZD2*, *SHROOM3*, *SOCS2*, *ACVR1B*, *STYK1*, *RHOB*, *FGD4*, *SSX2IP*, *CDC25A*, and *CHPT1* genes showed no significant increase following DHT treatment. qRT-PCR analysis of these genes showed that each was upregulated in the doxycycline treatment groups relative to the DHT treatment group, thus confirming the results obtained via microarray. Among these genes, *RHOB* seems to have the most robust upregulation by TC-AR, but not by DHT-bound FL-AR. This, together with its role in cell migration, was the basis for further examination.

### Knockdown of RHOB affects cell morphology and cell migration of LN/TC-AR cells under doxycycline treatments

**Figure 5:**
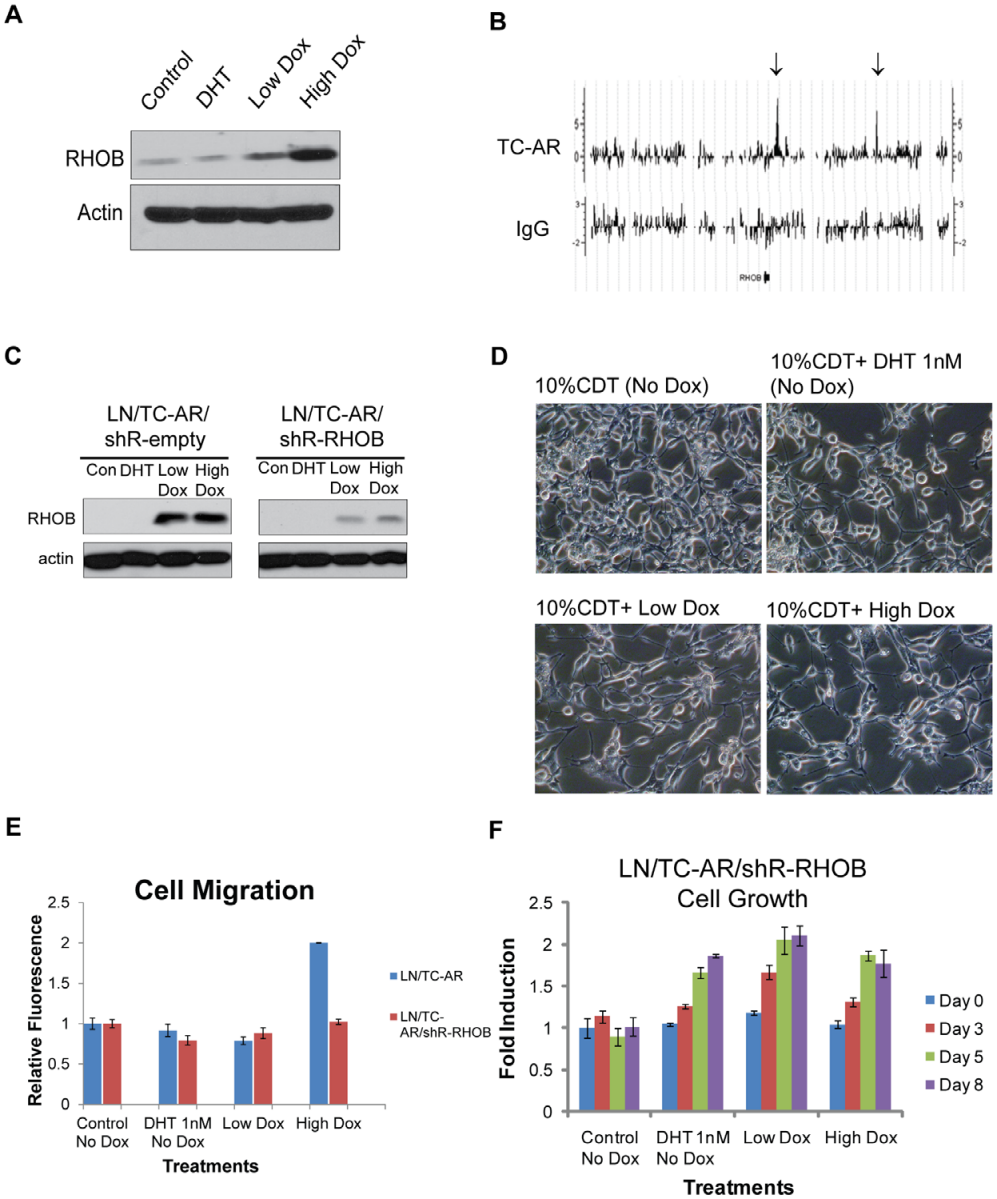
Knockdown of RHOB affects cell shape and cell migration following overexpression of TC-AR in LN/TC-AR/shR-RHOB. **A** LN/TC-AR cells were pre-cultured in hormone-depleted media for 48 hours then treated with Low Dox, High Dox, 1 nM DHT or vehicle for 24 hours. Whole cell lysates were harvested and subjected to western blot analysis. Membranes were probed with α-RHOB or α-actin. **B** Graphic representation of TC-AR binding sites 100 kb within the region of adjacent to RHOB gene. Arrows above show TC-AR binding sites (+3.8 kb and +47.5 kb downstream of TES). **C** Androgen-deprived LN/TC-AR/shR-empty and LN/TC-AR/shR-RHOB cells were treated with 1 nM DHT, Low Dox, High Dox or vehicle only. Whole cell lysates were harvested 48-hours post-treatment and subjected to western blot analysis. Membranes were probe with either α-RHOB (top) or α-actin (bottom). **D** Androgen-deprived LN/TC-AR/shR-RHOB cells were treated with 1 nM DHT, Low Dox, High Dox or vehicle. Images were acquired 48-hours post-treatment. **E** LN/TC-AR/shR-RHOB cells were pre-cultured in serum free media (SFM) for 24 hours then seeded to migration chambers with various treatments in the presence of SFM for an additional 48 hours after which time fluorescence was detected. Fold induction is relative to untreated control. Prior results for LN/TC-AR (from Figure 3B) are included for comparison. **F** MTT assay of LN/TC-AR/shR-RHOB in hormone depleted media following treatment with 1 nM DHT, Low Dox, High Dox or vehicle as control showing that DHT independent growth previously shown for LN/TC-AR (Figure 2D) is not inhibited by knockdown of RHOB. Bright field images were acquired with an Olympus microscope using 20× magnification.

*RHOB*, a small GTPase, is a member of the Ras-homologous (Rho) gene family, which plays a role in cell motility, apoptosis response and actin organization [22], [23]. The aforementioned microarray data showed the overexpression of RHOB is selectively induced by TC-AR. Western blot analysis confirmed the overexpression of RHOB protein in LN/TC-AR treated with Low and High Dox, but not in DHT treated cells without Dox induction (Figure 5A). Furthermore, ChIP to chip analysis revealed that under High Dox conditions, TC-AR is recruited to 3880 bp and 47521 bp downstream of transcription end site (TES) of *RHOB* (Figure 5B). Given the significant alterations of the cell morphology of LN/TC-AR upon doxycycline induction, we asked whether *RHOB* contributes to these changes.

To this end, shRNA was used to knock down RHOB expression in the LN/TC-AR cell line. Two new cell lines were established: LN/TC-AR/shR-RHOB in which shRNA targeting endogenous RHOB is constitutively expressed (TC-AR expression remains doxycycline dependent) and LN/TC-AR/shR-empty in which the shRNA sequence targeting RHOB has been removed. Western blot analysis of these lines revealed efficient knockdown of RHOB expression even following indirect induction with doxycycline via TC-AR-mediated upregulation (Figure 5C).

Images of LN/TC-AR/shR-RHOB cells were taken following treatment with 1 nM DHT, Low Dox or High Dox and culture in androgen depleted media for 48 hours. The shape of doxycycline-induced LN/TC-AR/shR-RHOB cells remained the same as DHT treated or control cells (Figure 5D). We then tested the effect of lower expression of RHOB on the migration of doxycycline-induced LN/TC-AR cells by performing a migration assay. The result showed that knockdown of RHOB negates the TC-AR overexpression mediated increase in migration of the LN/TC-AR cell line (Figure 5E). In order to test if knockdown of RHOB affects ADI growth of LN/TC-AR cells, an MTT assay was performed. LN/TC-AR/shR-RHOB cells were treated with 1 nM DHT, Low Dox, High Dox or vehicle as control and an MTT assay was completed on indicated days. Knockdown of RHOB did not affect the growth of DHT-treated cells, control cells or Low Dox-treated cells (Figure 5F). Thus, RHOB is likely to play a significant role in the morphological changes and migratory properties in LN/TC-AR cells, but not significantly involved in the proliferation of the cells.

## Discussion

It has been previously reported that simple overexpression of AR is sufficient to circumvent the normal androgen dependency of the LNCaP cell line [24] while severe overexpression of AR has deleterious effects [25]. Currently absent from the literature is a stable cell line in which physiologically relevant expression of AR forms are achieved. We have reported here the development of LN/TC-AR, a novel cell line in which TC-AR is inducible and titratable by doxycycline. This single cell line can closely recapitulate the balance between full-length and truncated forms of AR (Low Dox), mimic parental LNCaP (uninduced) or serve as a TC-AR “overexpression” line (High Dox) which has been useful to expedite/amplify subtle morphological and phenotypical characteristics. As a result, confounding variables present in studies which would otherwise utilize several cell lines are greatly reduced.

The truncated form of AR expressed in LN/TC-AR amounts to a “hybrid” receptor designed to be representative of naturally occurring C-terminally truncated AR forms. The stop codon is placed at Q641, the site of a non-sense mutation identified in a metastatic tumor taken from a patient whose disease had circumvented androgen deprivation therapy [26]. TC-AR also bears the exon 3 duplication identified in CWR22R*v1*, and, like AR^v567es^ (but not AR3/AR-V7), TC-AR retains the hinge domain and an intact nuclear localization signal. In order to ensure that these features did not confer unique activity relative to other possible forms of truncated AR, we tested a total of six similar forms of AR which varied in location of stop codon (R629, Q641 and V662) and the presence or absence of the exon 3 duplication and found DHT independent transactivation of each to be highly similar. We have also generated LN/TC-AR* which expresses an AR form with the same translational termination as TC-AR, but only a single exon 3. This receptor corresponds precisely to the aforementioned AR(Q641Stop) CRPC mutation [26]. Consistent with our previous report [2] and those of others [6], the addition of an extra exon3 does not seem to significantly affect the biological and biochemical properties of AR as transactivation, growth and cell shape studies for this cell line were consistent with results obtained using LN/TC-AR (Figure S2).

In establishing optimal expression of TC-AR, we observed the downregulation of endogenous AR following TC-AR induction. It has been previously reported that AR mRNA levels can be downregulated or upregulated by androgen depending on cell and tissue types [27] In order to limit the confounding effects of androgen withdrawal, the data presented here was obtained from assays completed in the presence of complete medium. It is worth noting that a similar pattern of repression was also observed in cells cultured in the absence of androgen. In addition to the expected decrease in AR protein levels following androgen withdrawal which is only exacerbated by induction of TC-AR, the abundance of AR mRNA decreased to approximately 50–75% that of uninduced controls (Figure S3). In LNCaP cells, studies indicate that androgen-mediated downregulation of AR mRNA levels is due to the decreased transcription of the AR gene [28], [29] as opposed to increased rate of mRNA turnover. There is no androgen response element (ARE) in the promoter of AR; however one study identified four AREs within the coding region of AR, but these AREs were reported to be involved exclusively in upregulation of AR mRNA [30]. An AR suppressor element (mARS) was identified in the mouse 5′UTR [31] with a similar element later identified in the human 5′UTR which was reported to be responsible for the decrease of AR promoter activity in androgen-dependent LNCaP cells [32].

Most recently, AR auto-repression was found to be due to the assembly of a FL-AR repressive complex containing lysine-specific demethylase 1 at the intron 2 enhancer [33]. We show here that AR autorepression is not limited to liganded FL-AR, but is effected by TC-AR as well. Unlike the similar AR^v567es^, co-immunoprecipitation assays revealed no detectable heterodimerization between FL-AR and TC-AR either in a transient transfection assay [2] or in LN/TC-AR (Figure 1E). While this result excludes repression by FL-AR/TC-AR heterodimers, a similar mode of repression by TC-AR homodimers is plausible and warrants further investigation within the context of LN/TC-AR and other truncated AR models.

At a minimalistic level, three criteria must be met for a truncated form of AR to be considered a potential surrogate for DHT-bound FL-AR in the absence of DHT: a) it must retain the ability to localize to the cell nucleus, b) facilitate transcription of AR target genes and, c) “rescue” the effect of growth inhibition. Immunostaining of LN/TC-AR following induction with Low Dox reveals that TC-AR predominantly localizes to the cell nucleus in the absence of DHT. Although a commonly used reporter assay (transient transfection of pPSA-luciferase) served as a first screen of LN/TC-AR, that assay is not representative of gene activation in chromatin. Via ChIP analysis of LN/TC-AR we have shown *KLK3* promoter occupancy by TC-AR and RNAP2 in the absence of DHT. *KLK3* was also found by microarray analysis to be upregulated by TC-AR. Together, these results demonstrate that TC-AR is able to facilitate transcription at an endogenous AR-regulated promoter in the absence of DHT.

Lastly, we have shown that TC-AR expression is sufficient to confer ADI growth to the normally AD LNCaP cell line and that this effect is tethered tightly to the physiologically relevant expression of TC-AR. LN/TC-AR induced with 3 ng/mL doxycycline fail to grow in conditions of androgen deprivation while cells induced with 5–10 ng/mL doxycycline show ADI proliferation inversely proportional to doxycycline concentration. This is consistent with recent reports in which the growth-promoting effects of some AR splice variants were mediated through FL-AR [15] given the reduction in FL-AR following overexpression of TC-AR. In a subsequent assay, however, it was determined that the AR inhibitor MDV3100 does not repress ADI growth of LN/TC-AR when both AR forms are expressed (not shown).

Although these basic biochemical properties of TC-AR closely approximate DHT-bound endogenous AR in LNCaP, microarray analysis of regulated genes has shown that, while there is significant overlap between the two as would be expected, there also exist differences in that pool of genes. One explanation for this difference could be the differential assembly of cofactors. Common to both TC-AR and endogenous full-length AR is the N-terminal domain (NTD) which serves as the primary binding region for cofactors such as SRC-1 and GRIP1 [34] and contains activation function-1a (AF-1a) and AF-1b [35] which are the major AF regions of AR. Despite retention of these domains, it is unclear if and how the loss of the LBD affects cofactor recruitment and/or binding in TC-AR.

Comparisons of existing transcriptome studies can be difficult given variances in model systems, growth conditions, sample preparation and the wide selection of platforms for such analyses. This is evident in a comparison of the data acquired here with those of two prior studies in which the transcriptome of a similarly truncated AR form, denoted either AR3 [8] or AR-V7 [36] was interrogated. Comparison of our microarray data and data generated in those studies shows very little commonality and qRT-PCR analysis confirmed microarray data showing that three genes of focus in those studies (AKT1, CDC20 & UBE2C) are not upregulated following induction of TC-AR in the LN/TC-AR model (Figure S4). However, the data acquired here is consistent with a third study in which the transcriptome of AR^v567es^ was assayed [11]. Among the 26 top-upregulated genes identified in that study, 15 were confirmed here with three upregulated by FL-AR only (*BRP44*, *DHCR24* & *HERC3*), seven upregulated by TC-AR only (*IDI*, *ORM1*, *SSR3*, *SC4MOL*, *SEC24D*, *SGK1* & *STEAP4*) and five common to the transcriptomes of both FL-AR and TC-AR (*ELL2*, *MBOAT2*, *SLC45A3*, *STK39* & *TMPRSS2*). Also common to both TC-AR and AR^v567es^ is the hinge region and intact nuclear localization signal. This region is potentially a source of variation in these studies as it is not present in AR3/AR7 which instead terminates with amino acids encoded by a cryptic exon.

Early in our characterization of LN/TC-AR, we found that cell shape changed rapidly following severe overexpression of TC-AR and at a much slower rate and to a slightly lesser degree following induction of physiologically relevant levels of TC-AR. As deregulated cell adhesion of tumor cells, often first observed as a change in cell shape, can lead to cell detachment and promote cell invasion [37], we hypothesized that migration of LN/TC-AR may be affected by modulation of TC-AR expression. We found that LN/TC-AR displayed a two fold increase in migration following overexpression of TC-AR and that this result correlated with the striking change to cell shape. We hypothesized that FGD4, RHOB or SSX2IP, all determined to be upregulated in microarray and western blot studies and known to be involved in cell adhesion, cell shape or cell motility, may be involved in this phenotypic change. We generated LN/TC-AR/shR-FGD4, LN/TC-AR/shR-RHOB and LN/TC-AR/shR-SSX2IP and, although all lines showed significant knockdown of either FGD4, RHOB or SSX2IP, only LN/TC-AR/shR-RHOB negated the cell shape changes caused by overexpression of TC-AR (data not shown). Consistent with the observation of cell shape, a repeat of the migration assay, this time using LN/TC-AR/shR-RHOB in place of LN/TC-AR, showed that overexpression of TC-AR failed to cause an increase in migration.

Rho GTPase, RHOA, RHOB and RHOC are known for regulation of the cytoskeleton, cell motility and cell cycle [22], [38]. Based on our microarray data, the expression of RHOA and RHOC were not significantly changed by the induction of TC-AR (data not shown). However, we found that the expression of RHOB mRNA and protein levels were significantly elevated in doxycycline-treated LN/TC-AR cells. While the function of RHOB in disease progression is complex and context dependent [38]–[42], we have shown here that RNAi-mediated silencing of RHOB inhibited the change of cell shape as well as cell migration of LNCaP expressing TC-AR. Similarly, a recently published report has shown that overexpression of RHOB in the ADI CaP line DU145 enhances cell migration [43]. Lastly, RHOB has been shown to be upregulated and negatively affect cell proliferation of some types of cancer cells [23]. MTT analysis of the growth of LN/TC-AR/shR-RHOB supports this role of RHOB as the high degree of cell death following significant overexpression of TC-AR in LN/TC-AR is not observed in LN/TC-AR/shR-RHOB.

To summarize, we present here a new cell line to study the effect of truncated AR on the progression of prostate cancer to an androgen depletion independent disease. As part of our initial characterization of one such representative form of truncated AR, we have shown that, similar to DHT-bound endogenous full-length AR, the ligand independent truncated AR retains the ability to translocate to the cell nucleus where it is able to bind AREs within chromatin and facilitate transcription of a host of androgen regulated genes. Beyond this similarity, we have identified differences in the targets of transcriptional upregulation with one such difference being the upregulation of RHOB, a gene whose expression is not influenced by DHT-bound full-length AR. As validation of the usefulness of this model cell line, we have shown the upregulation of RHOB to be involved in the increased motility and morphological changes evident in androgen depletion independent growth conferred by the truncated AR.

## Supporting Information

Figure S1 LN/TC-AR cells were grown in the presence of hormone depleted media and treated with 4.5 ng/mL of doxycycline (low dox) or left untreated as control for 6 days. Media and doxycycline were refreshed every 3 days. Bright field images were acquired with an Olympus microscope using 20× magnification.
(TIF)

Figure S2 A Western blot showing TC-AR* levels of LN/TC-AR* lines. Doxycycline concentrations are shown above the membrane image. Membrane were probed with α-AR (PG-21) (primary) followed by α-mouse (secondary). Simultaneous with α-AR (PG-21), each membrane was also probed with α-actin (primary) as a control for loading. B Morphology of LN/TC-AR* cell line following induction of TC-AR*. LN/TC-AR* cells were grown in the presence of hormone depleted media and treated with various concentrations of doxycycline (listed immediately above images). At 48-hours post-treatment representative images of each sample group were acquired. C Androgen independent growth of the LN/TC-AR* cell line. Cell count assay showing the growth of LN/TC-AR*. Cells were cultured in androgen-depleted medium that was supplemented with either 1 nM DHT, low dox, high dox or vehicle only. At the designated time points, total cells per well were determined via Countess® Automated Cell Counter. D Luciferase assay showing androgen-independent activation of TC-AR* Luciferase assay showing that TC-AR*-mediated transactivation is not dependent upon androgen within the context of the LN/TC-AR* cell line. LN/TC-AR* cells were co-transfected with pPSA6.0-luc and pH 48-ren in hormone depleted media and treated with low concentrations of doxycycline (listed below the graph) and either 1 nM DHT or vehicle as control for 24 hours. Fold induction is reported relative to uninduced LN/TC-AR* cells grown in the absence of DHT.
(TIF)

Figure S3 AR mRNA in LN/TC-AR cultured in androgen depleted medium decreases following induction of TC-AR. Graph shows data obtained from two separate microarray probe clusters targeting the LBD and 3′UTR of AR. Neither region is present in TC-AR thus ensuring analysis of only endogenous AR mRNA. Cluster 211110_s_at contains a mix of 11 probes spanning nucleotides 3386–3950 (Exon 5 to 3′UTR) and cluster 211621_at contains a mix of 11 probes spanning nucleotides 4010–4251 (3′UTR). All samples for microarray analysis were prepared as described in the main text. Nucleotide positions are based on NCBI Reference Sequence NM_000044.3.
(TIF)

Figure S4 Quantitative Real Time PCR Analysis of AKT1, CDC20 & UBE2C. qRT-PCR was performed as described in Materials and Methods using primers shown in Table S3. Results show no significant upregulation of these three genes following induction of TC-AR in the LN/TC-AR cell line.
(TIF)

Table S1 Genes up-regulated by DHT, Low Dox and High Dox treatments
(XLSX)

Table S2 Genes up-regulated by Low Dox and High Dox treatments
(XLSX)

Table S3 Sequences of primers for q-RT-PCR
(XLSX)
