# Supplementary figures and images for: Modeling Truncated AR Expression in a Natural Androgen Responsive Environment and Identification of RHOB as a Direct Transcriptional Target
Source: PLoS One. 2012 Nov 29;7(11):e49887. doi: 10.1371/journal.pone.0049887 (PMC3510170; doi:10.1371/journal.pone.0049887)

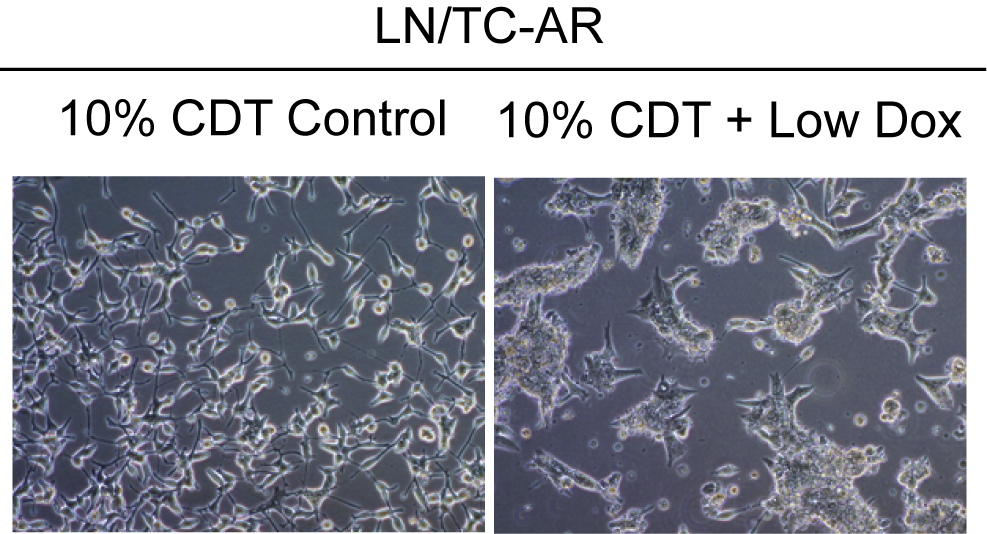

Supplement: Figure S1 — LN/TC-AR cells were grown in the presence of hormone depleted media and treated with 4.5 ng/mL of doxycycline (low dox) or left untreated as control for 6 days. Media and doxycycline were refreshed every 3 days. Bright field images were acquired with an Olympus microscope using 20× magnification. (TIF) [file pone.0049887.s001.tif]

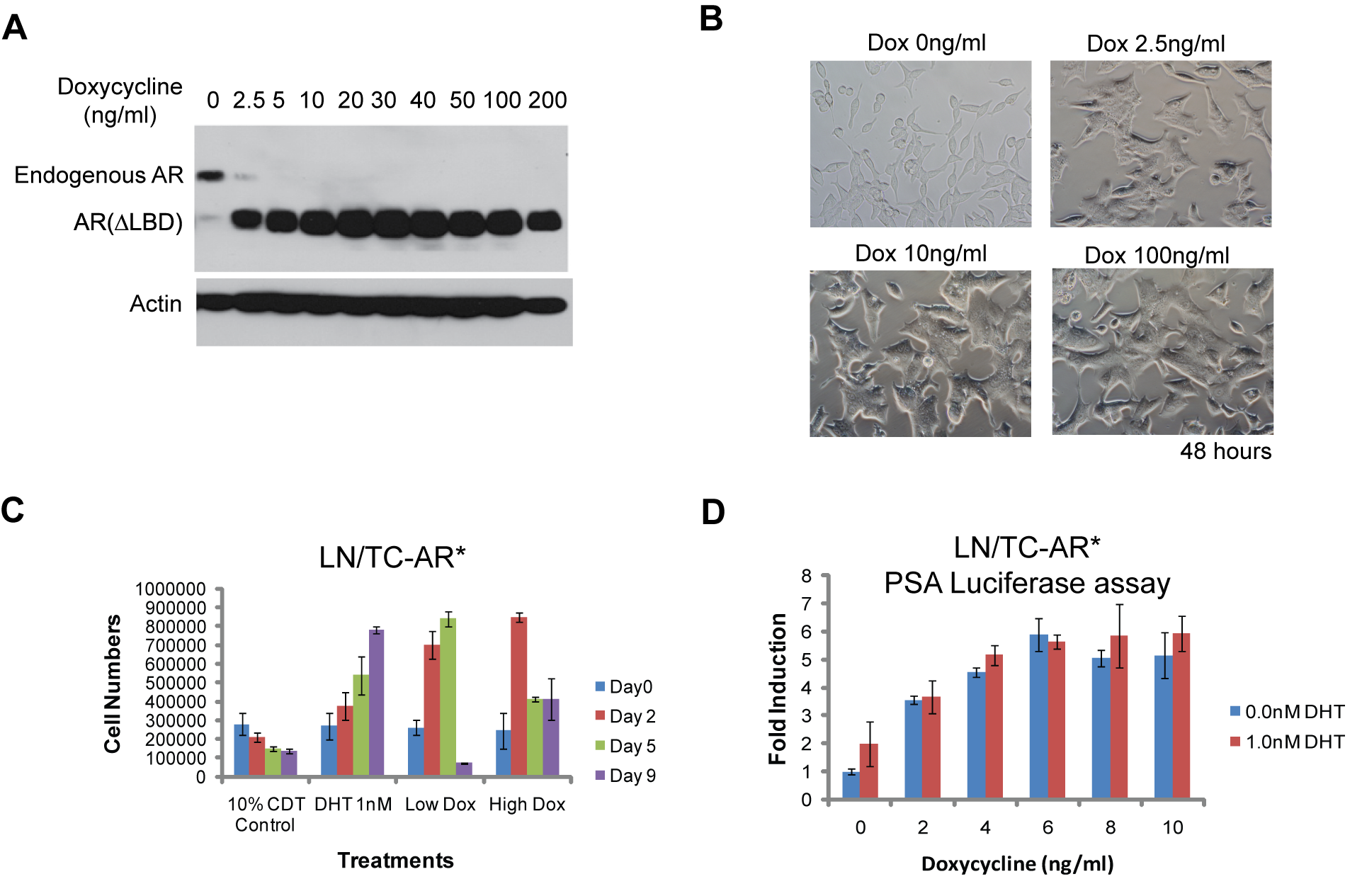

Supplement: Figure S2 — A Western blot showing TC-AR* levels of LN/TC-AR* lines. Doxycycline concentrations are shown above the membrane image. Membrane were probed with α-AR (PG-21) (primary) followed by α-mouse (secondary). Simultaneous with α-AR (PG-21), each membrane was also probed with α-actin (primary) as a control for loading. B Morphology of LN/TC-AR* cell line following induction of TC-AR*. LN/TC-AR* cells were grown in the presence of hormone depleted media and treated with various concentrations of doxycycline (listed immediately above images). At 48-hours post-treatment representative images of each sample group were acquired. C Androgen independent growth of the LN/TC-AR* cell line. Cell count assay showing the growth of LN/TC-AR*. Cells were cultured in androgen-depleted medium that was supplemented with either 1 nM DHT, low dox, high dox or vehicle only. At the designated time points, total cells per well were determined via Countess® Automated Cell Counter. D Luciferase assay showing androgen-independent activation of TC-AR* Luciferase assay showing that TC-AR*-mediated transactivation is not dependent upon androgen within the context of the LN/TC-AR* cell line. LN/TC-AR* cells were co-transfected with pPSA6.0-luc and pH 48-ren in hormone depleted media and treated with low concentrations of doxycycline (listed below the graph) and either 1 nM DHT or vehicle as control for 24 hours. Fold induction is reported relative to uninduced LN/TC-AR* cells grown in the absence of DHT. (TIF) [file pone.0049887.s002.tif]

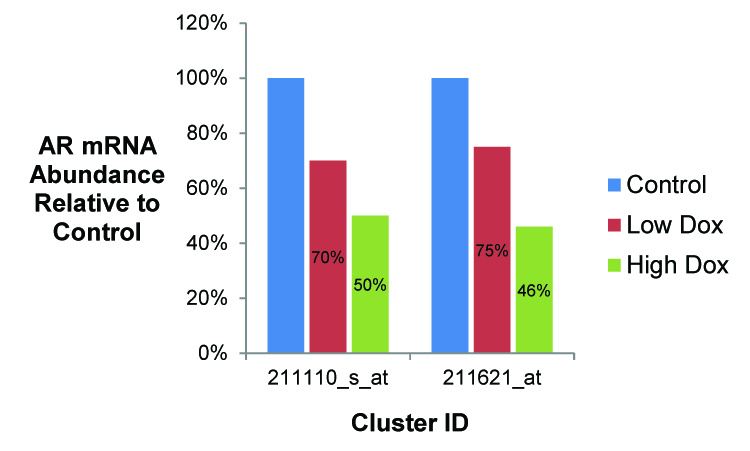

Supplement: Figure S3 — AR mRNA in LN/TC-AR cultured in androgen depleted medium decreases following induction of TC-AR. Graph shows data obtained from two separate microarray probe clusters targeting the LBD and 3′UTR of AR. Neither region is present in TC-AR thus ensuring analysis of only endogenous AR mRNA. Cluster 211110_s_at contains a mix of 11 probes spanning nucleotides 3386–3950 (Exon 5 to 3′UTR) and cluster 211621_at contains a mix of 11 probes spanning nucleotides 4010–4251 (3′UTR). All samples for microarray analysis were prepared as described in the main text. Nucleotide positions are based on NCBI Reference Sequence NM_000044.3. (TIF) [file pone.0049887.s003.tif]

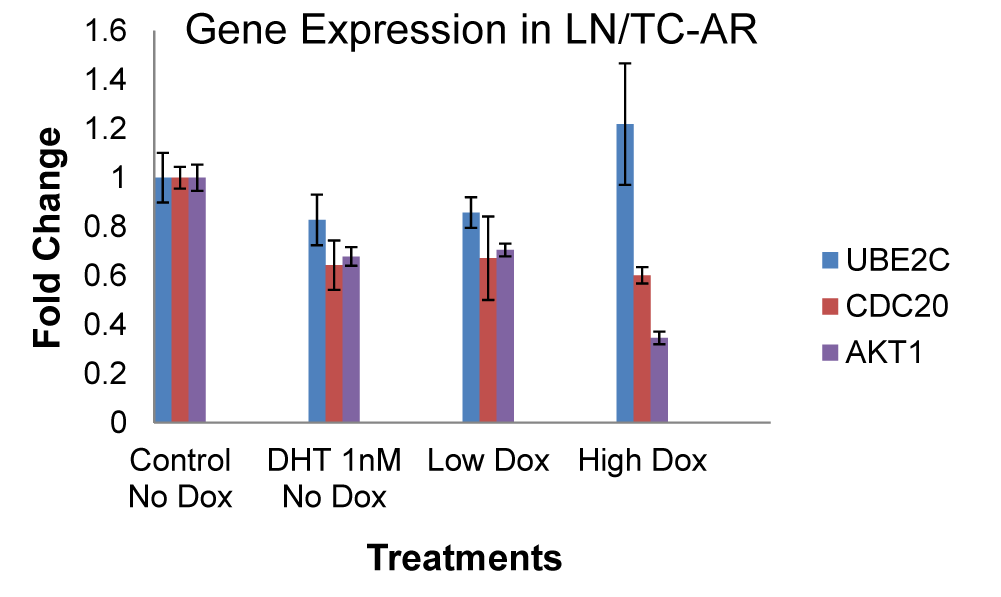

Supplement: Figure S4 — Quantitative Real Time PCR Analysis of AKT1, CDC20 & UBE2C. qRT-PCR was performed as described in Materials and Methods using primers shown in Table S3. Results show no significant upregulation of these three genes following induction of TC-AR in the LN/TC-AR cell line. (TIF) [file pone.0049887.s004.tif]
